# Supplementary material for: Content-rich biological network constructed by mining PubMed abstracts
Source: BMC Bioinformatics. 2004 Oct 8;5:147. doi: 10.1186/1471-2105-5-147 (PMC528731; doi:10.1186/1471-2105-5-147)
Supplement: Additional File 5 — The original Chilibot query results of the term "long-term potentiation (LTP)" and 22 other terms, limiting the latest references analyzed to the years 1990, 1995, 2000, and 2004. [file 1471-2105-5-147-S5.bz2 › chilibotAdditionalFile5/ltp2004/html/PKC_ACTIN.html]

 


 **PKC** and **ACTIN** 
  
Found 541 abstracts in PubMed,  **30 abstracts were retrieved and analyzed**.  


---

 Search Google  |
 PDF files only 
|  EDU domain only 

---

**Interactive relationship** (e.g. stimulation, inhibition, etc)

- 3 The ability to inhibit the dynamics of NF kappaB and F  **actin**  cytoskeletal disassembly is a novel mechanism not previously attributed to the classical subfamily of  **PKC**  isoforms.  Ref: 14602581 Am J Physiol Cell PhysiolAm J Physiol Cell Physiol, 1999
- C Stably inhibiting native  **PKC**  beta1 99% substantially inhibited all measures of EGF mediated protection against NF kappaB activation and F  **actin**  dysfunction.  Ref: 14602581 Am J Physiol Cell PhysiolAm J Physiol Cell Physiol, 1999
- TGFbeta1 induced expression of alpha SM  **actin**  was inhibited in a dose dependent manner by treating cells with a  **PKC**  inhibitor, calphostin C, and was abolished by depleting PKCalpha with antisense PKCalpha oligodeoxynucleotides.  Ref: 12967633 J Mol Cell Cardiol, 2003
- **PKC**  beta 1 Isoform Activation is Required for EGF Induced NF kappa B Inactivation and I kappa B alpha Stabilization and Protection of F  **Actin**  Assembly and Barrier Function in Enterocyte Monolayers.  Ref: 14602581 Am J Physiol Cell PhysiolAm J Physiol Cell Physiol, 2003
- In addition in vitro studies Biochemistry 39 2000 271 have suggested that all  **PKC**  isoforms bind to  **actin**  during the process of activation of the enzyme.  Ref: 14499623 Exp Cell Res, 2003
- These results suggest that the  **actin**  network is not involved in the initial steps of the  **PKC**  activation process in living nerve cells.  Ref: 14499623 Exp Cell Res, 2003
- Previous studies have shown that n alkanols have biphasic chain length dependent effects on protein kinase C  **PKC**  activity induced by association with membranes or with filamentous  **actin**  Slater, S.  Ref: 14556642 Biochemistry, 2003
- In optic tract, AA mediates NCAM and L1 stimulation of axon growth by activating presynaptic protein kinase C  **PKC**  to phosphorylate GAP 43 and stabilize F  **actin** , and, if present in tectum, this growth control pathway could be modulated by postsynaptic activation.  Ref: 14750146 J Neurobiol, 2004
- Our results allow us to speculate that a preinflammatory substance, such as LPA, interleukin 1 beta, and bradykinin, augments MARCKS phosphorylation in a novel signal transduction pathway besides the  **PKC**  involved one, and thereby induces the release of a neurotransmitter through a reorganization of  **actin**  containing microfilaments at the cell periphery, the so called active zone.  Ref: 14501149 J Pharmacol Sci, 2003
- **Actin**  is a major substrate for protein kinase C  **PKC**  and  **PKC**  is considered a modulator of the  **actin**  network.  Ref: 14499623 Exp Cell Res, 2003

**Parallel relationship** (e.g. studied together, co-existance, homology, etc.)

- Escherichia coli, the most common Gram negative bacterium that causes meningitis in neonates, invades human brain microvascular endothelial cells HBMEC by rearranging host cell  **actin**  via the activation of phosphatidylinositol 3 kinase PI3K and  **PKC**  alpha.  Ref: 12952950 J Biol Chem, 2003
- We inhibited Src PP2, FAK small RNA interference, SiRNA, or FRNK transfection, MEK PD98059,  **PKC**  calphostin C, and  **actin**  destabilization phalloidin.  Ref: 14699482 Gastroenterology, 2004
- Together, these results indicate that CD151 is involved in epithelial cell cell adhesion as a modulator of  **PKC**  and Cdc42 dependent  **actin**  cytoskeletal reorganization.  Ref: 14557253 J Cell Biol, 2003
- In this study, we further demonstrate that DcR3 is able to induce  **actin**  reorganization and enhance the adhesion of monocytes and THP 1 cells by activating multiple signaling molecules, such as protein kinase C  **PKC** , phosphatidylinositol 3 kinase PI3K, focal adhesion kinase FAK and Src kinases.  Ref: 14697332 Exp Cell Res, 2004
- Myristoylated alanine rich C kinase substrate MARCKS is an  **actin**  binding protein whose function may be regulated by the phosphorylation of multiple sites, in which the phosphorylation site domain PSD is recognized to have three or four  **PKC**  dependent sites.  Ref: 14501149 J Pharmacol Sci, 2003
- The  **actin**  corrected ratios for all other  **PKC**  isozymes, alpha Day 4 = 0.93 0.16, Day 10 = 0.97 0.09, betaI Day 4 = 0.54 0.073, Day 10 = 0.48 0.74, and micro Day 4 = 0.21 0.042, Day 10 = 0.21 0.38 were not different at these 2 days of the cycle.  Ref: 14522831 Biol ReprodBiol Reprod, 2003
- When PC12 cells were treated with either latrunculin B, which impairs  **actin**  polymerization, or phalloidin, which stabilizes  **actin**  filaments, we observed a significant reduction of the calcium i response revealed by Fura 2 fluorescence, while the  **PKC**  conformational changes followed by Fim 1 fluorescence were unaffected.  Ref: 14499623 Exp Cell Res, 2003
- **PKC**  and cytoskeletal elements pivotal to axonogenesis beta tubulin and  **actin**  during perinatal development of mouse retina.  Ref: 14519497 Brain Res Dev Brain Res, 2003
- To investigate the molecular processes underlying EGF protection against oxidant disruption, we hypothesized that EGF induced activation of  **PKC**  beta1 prevents oxidant induced activation of NF kappaB and the consequences of NF kappaB activation, namely F  **actin**  disassembly and barrier dysfunction.  Ref: 14602581 Am J Physiol Cell PhysiolAm J Physiol Cell Physiol, 2003
- In this study, we investigated the effects of the  **actin**  cytoskeletal architecture on NO induced dedifferentiation, apoptosis, cyclooxygenase COX 2 expression, and prostaglandin E2 production in articular chondrocytes, with a focus on ERK 1 2, p38 kinase, and  **PKC**  signaling.  Ref: 12907684 J Biol Chem, 2003
- Peptide loading also resulted in a significant inhibition of phorbol ester induced adducin Ser662 phosphorylation, an intracellular monitor of  **PKC**  kinase activity, ERK1 2 activation, and Ser789 phosphorylation of the  **actin**  binding protein caldesmon.  Ref: 12969891 Am J Physiol Heart Circ Physiol, 2004
- CD151 regulates epithelial cell cell adhesion through  **PKC**  and Cdc42 dependent  **actin**  cytoskeletal reorganization.  Ref: 14557253 J Cell Biol, 2003
- While Vav1 contributes to Rac dependent reorganization of the  **actin**  cytoskeleton, activated PLCgamma1 generates the protein kinase C  **PKC**  activator diacylglycerol.  Ref: 14656980 FASEB J, 2003
